# Clinical Use of the Edmonton Obesity Staging System for the Assessment of Weight Management Outcomes in People with Class 3 Obesity

**DOI:** 10.3390/nu14050967

**Published:** 2022-02-24

**Authors:** Raymond Kodsi, Ritesh Chimoriya, David Medveczky, Kathy Grudzinskas, Evan Atlantis, Abd A. Tahrani, Nic Kormas, Milan K. Piya

**Affiliations:** 1 South Western Sydney Metabolic Rehabilitation and Bariatric Program (SWS MRBP), Camden and Campbelltown Hospitals, Camden, NSW 2570, Australia; ray.kodsi@gmail.com (R.K.); kathy.grudzinskas@health.nsw.gov.au (K.G.); nic.kormas@health.nsw.gov.au (N.K.); 2 School of Medicine, Western Sydney University, Campbelltown, NSW 2560, Australia; r.chimoriya@westernsydney.edu.au (R.C.); davidmedveczky@gmail.com (D.M.); 3 School of Health Sciences, Western Sydney University, Campbelltown, NSW 2560, Australia; e.atlantis@westernsydney.edu.au; 4 Discipline of Medicine, Nepean Clinical School, Faculty of Medicine and Health, The University of Sydney, Nepean, NSW 2747, Australia; 5 Institute of Metabolism and Systems Research, University of Birmingham, Birmingham B15 2TT, UK; abd.tahrani@nhs.net; 6 Centre for Endocrinology, Diabetes and Metabolism (CEDAM), Birmingham Health Partners, Birmingham B15 2TT, UK; 7 Department of Diabetes and Endocrinology, University Hospitals Birmingham NHS Trust, Birmingham B15 2TT, UK

**Keywords:** weight management, class 3 obesity, obesity staging

## Abstract

We aimed to assess weight loss and metabolic outcomes by severity of weight-related complications following an intensive non-surgical weight management program (WMP) in an Australian public hospital. A retrospective cohort study of all patients aged ≥18 years with body mass index (BMI) ≥ 40 enrolled in the WMP during March 2018–March 2019 with 12-month follow-up information were stratified using the Edmonton Obesity Staging System (EOSS). Of 178 patients enrolled in the WMP, 112 (62.9%) completed at least 12 months’ treatment. Most patients (96.6%) met EOSS-2 (56.7%) or EOSS-3 (39.9%) criteria for analysis. Both groups lost significant weight from baseline to 12 months; EOSS-2: 139.4 ± 31.8 kg vs. 131.8 ± 31.8 kg (*p* < 0.001) and EOSS-3: 141.4 ± 24.2 kg vs. 129.8 ± 24.3 kg (*p* < 0.001). After adjusting for baseline age, sex and employment status, mean weight loss was similar but a greater proportion of EOSS-3 achieved >10% weight loss compared to EOSS-2, (40% vs. 15.9%, *p* = 0.024). Changes in metabolic parameters including HbA1c, BP and lipids did not differ between EOSS-2 and 3. Despite increased clinical severity, adult patients with class 3 obesity achieved clinically meaningful weight loss and similar improvements in metabolic parameters compared to patients with less severe complications after 12 months in an intensive non-surgical WMP.

## 1. Introduction

Obesity is associated with significantly increased mortality [1,2] and is often defined in clinical practice by a body mass index (BMI) greater than or equal to 30 in a White European population [3,4,5]. Despite the usefulness, simplicity and broad applicability of the BMI, it is identified as a poor indicator of body fat as a health issue [6]. While the addition of waist circumference (WC) or waist to hip ratio (WHR) is more predictive of mortality and morbidity than BMI alone, these measures remain suboptimal predictors of mortality [5,6,7,8,9,10]. Furthermore, anthropometric measures of obesity provide no information on physical function, quality of life or co-morbid conditions [11]. 

The Edmonton Obesity Staging System (EOSS) is a widely used and validated staging system based on weight-related health impairments among individuals with obesity. The EOSS categorises obesity severity based on the impact of obesity-related complications on medical, physical and psychological health [11]. Table 1 outlines how patients with obesity based on anthropometric measures are stratified into classes 0 to 4 based on the most severe of their obesity-related co-morbidities, with stage 0 reflecting no obesity-related issues and stage 4 reflecting end-stage complications of obesity [12]. Increasing EOSS severity has been shown to correlate with mortality better than BMI [5,9,13], and has been linked to increased health service use, polypharmacy and less weight loss [6,13]. A previous technology assessment by the Institute for Clinical and Economic Review in 2015 recommended that health systems should use obesity classification systems, such as EOSS instead of BMI, to more effectively target treatment resources [14].

Treatment of severe obesity is challenging and usually requires comprehensive lifestyle interventions and multidisciplinary medical management, including pharmacotherapy and bariatric surgery where appropriate [15]. Bariatric surgery is currently the most effective treatment option and results in sustained long term weight loss, although barriers to access have been reported in many countries globally [16]. While approximately 1 million Australians meet criteria for bariatric surgery, only around 20,000 procedures are performed per year in Australia, and only 2000 patients have access to publicly funded specialist obesity services [15,17,18], with much fewer having access to publicly funded bariatric surgery. National and international guidelines recommend referral to specialist weight management programs or bariatric surgery for patients with BMI ≥ 40 or patients with BMI ≥ 35 with a weight-related co-morbidity [5,19,20]. However, due to limited resources, many weight management programs use BMI-based criteria to restrict entry only to patients with higher BMIs [15], and selection criteria may vary by centre [21]. Despite its widespread use, baseline BMI on its own may not predict favourable outcomes for patients [22] and does not measure “health”. The EOSS may be a better alternative for patient prioritisation and prognostication for these interventions as it provides information on the patients’ biopsychosocial health rather than solely anthropometrics [13,21,23,24,25,26,27]. It is important to ascertain the effectiveness of weight loss interventions across different grades of severity of obesity and its complications. We hypothesised that increasing EOSS severity would result in less weight loss due to increasing limitations on physical activity as well as ability to follow suggested dietary recommendations, with greater comorbidities.

In this study, we aimed to assess weight loss and metabolic outcomes by the Edmonton Obesity Staging System (EOSS) following an intensive non-surgical weight management program (WMP) in an Australian public hospital.

## 2. Methods

### 2.1. Study Design

This study was a retrospective cohort study of all new participants enrolled in a multidisciplinary weight management program in a Sydney hospital between March 2018 and March 2019. All patients included in the study had attended at least one medical appointment. Inclusion criteria for this clinic were age ≥18 years and BMI ≥ 40, with at least one obesity-related co-morbidity, most often T2DM or NAFLD. The multidisciplinary team consisted of endocrinologists, a gastroenterologist, dietitians, a psychiatrist, psychologists, physiotherapists and a specialist nurse. The multidisciplinary team provided individualised care to each patient, which included a combination of one or more lifestyle intervention, behavioural modification, dietary advice and pharmacotherapy. Some patients in the cohort were interested in bariatric surgery, some were not, and some were undecided. None of the patients underwent bariatric surgery during the 12-month period of this study. All patients were seen by a physician, dietitian and psychologist. The weight management program has been described in detail previously [28,29,30,31]. Dietary advice was personalised, which included but was not limited to advice for a 500 Kcal reduced diet, partial meal replacement and/or a low-calorie diet. Dietary advice was based on patient preference and weight trajectory. Depending on the clinical need, patients were reviewed every 6–12 weeks and had access to a physiotherapist, clinical nurse consultant, gastroenterologist or psychiatrist as required. 

### 2.2. Data Collection

Data were collected at baseline and 12 months for anthropometry, co-morbidities, medication use and medication doses from patient electronic medical records and paper notes. All patients who initially enrolled in the program were included in baseline data collection and analysis. Data on weight, medical comorbidities, medications and blood tests were collected from routine clinical data at baseline and 12 months of follow-up, where available. BMI was calculated by dividing weight in kilograms by square of height in meters. Patients who did not follow up at 12 months were not included in the 12-month analysis. All analyses of glycaemic outcomes and diabetes medication use were limited to patients with T2DM at baseline. Patients were stratified into an EOSS score by a single assessor retrospectively, according to the criteria proposed by Sharma et al. and Canning et al. [11,12]. A patient’s score was defined according to the highest of the mental, physical and function scores present. 

### 2.3. Data Analysis

Data were analysed using the Statistical Package for Social Sciences, Version 27 (SPSS for MacOS, SPSS Inc., Chicago, IL, USA). Normality for continuous variables was determined by Shapiro–Wilk test. Parametric data were analysed using independent t-test and results were expressed as mean ± SD. Difference between baseline and 12-month values of parametric data within the same group was analysed using paired t-test. Percentage change in continuous variables were analysed using ANCOVA, using change in raw value as the dependent variable and the baseline variable as the co-variate. Data were adjusted for age, sex and employment status at baseline (considering baseline difference in the characteristics between two groups) using a regression analysis; change in mean and SD at 12 months were then expressed as percentage of the baseline value. Non-parametric data were analysed using Mann–Whitney U and Wilcoxon Sign Rank Tests. Categorical variables were analysed using Pearson’s chi-square test and the association to a categorical outcome was adjusted for age and sex at baseline using logistic regression. 

### 2.4. Ethics

This study was approved by the South West Sydney Local Health District Research Ethics Committee as a quality improvement project (Reference: CT22_2018). This study was conducted in accordance with the Declaration of Helsinki.

## 3. Results

### 3.1. Baseline Characteristics

**Figure 1:**
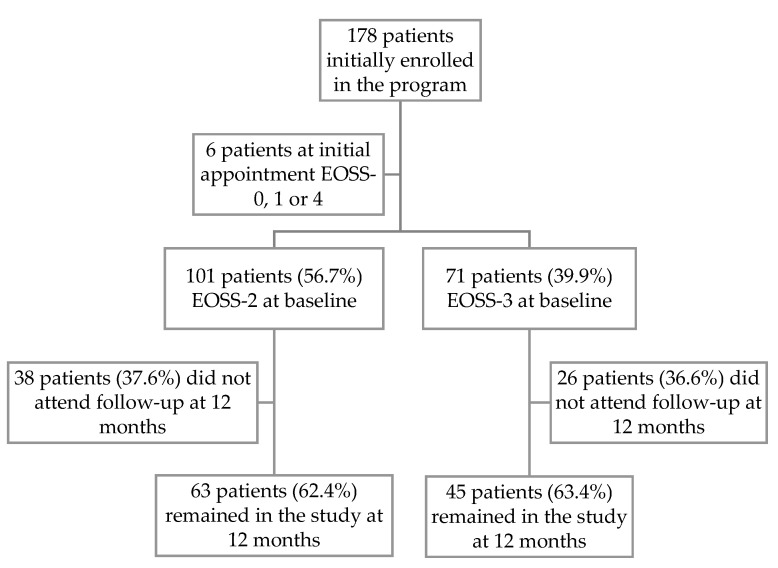
Flow diagram of subjects enrolled in the multidisciplinary weight management program at baseline and 12 months, by initial EOSS group.

Of the 178 new patients enrolled into the program between March 2018 and March 2019, 112 patients (62.9%) remained enrolled in the program at 12 months. Almost all patients were either EOSS-2 (*n* = 101, 56.7%) or EOSS-3 (*n* = 71, 39.9%) at baseline. Given the small numbers in other EOSS groups with EOSS-0 (1.1%), EOSS-1 (0.6%) and EOSS-4 (1.7%), subsequent analysis was limited to the 172 patients who were initially EOSS-2 or EOSS-3 at baseline (Figure 1). There was no difference in dropout rates between EOSS-2 and EOSS-3 groups (62.4% vs. 63.4%). 

### 3.2. Weight Loss and Impact on Metabolic Outcomes in EOSS-2 and 3

As shown in Table 2, there was no significant difference in baseline weight between the EOSS groups. At baseline, EOSS-2 patients were significantly younger, and more were in paid employment than EOSS-3. The EOSS-2 group also had a lower prevalence of T2DM, hypertension and dyslipidaemia. Although glycaemic control among patients with T2DM was comparable in both EOSS-2 and EOSS-3 groups, EOSS-3 patients had diabetes for longer and more were on insulin therapy. Similarly, although there were no differences in systolic blood pressure or lipid profile, EOSS-3 patients had a greater medication burden for hypertension and dyslipidaemia compared to the EOSS-2 group. There was also no difference between EOSS groups in the mean levels of calcium, phosphate, magnesium, folate, vitamin B12 or vitamin D.

### 3.3. Comparing Weight Loss and Metabolic Outcomes in EOSS-2 vs. EOSS-3

**Figure 2:**
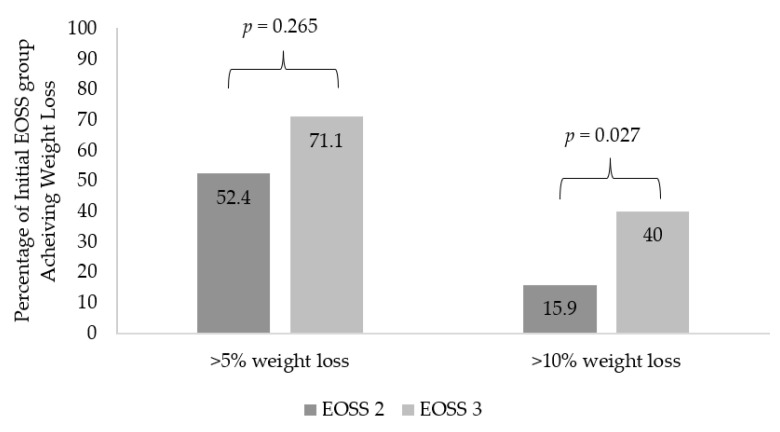
Proportion of each EOSS group achieving weight loss milestones. After adjusting for baseline age, sex and employment status, adjusted *p*-values were *p* = 0.265 and *p* = 0.027 for >5% and >10% weight loss, respectively.

As seen in Table 3, both EOSS-2 and EOSS-3 groups lost a significant amount of weight at 12 months. As shown in Table 4, compared to the EOSS-2 group, percentage weight loss was greater in the EOSS-3 group (5.7 ± 5.3% vs. 8.2 ± 7.3%, *p* = 0.047). However, this significance was lost after adjusting for baseline age, sex and employment status (*p* = 0.203). Moreover, as seen in Figure 2, compared to the EOSS-2 group, a higher proportion of EOSS-3 patients were able to lose greater than 5% (52.4% vs. 71.1%, *p* = 0.05) or 10% (15.9% vs. 40.0%, *p* = 0.005) of their body weight. This remained significant after adjusting for baseline age for 10% weight loss (*p* = 0.024), but significance was lost for 5% weight loss (*p* = 0.257).

In patients who had T2DM at baseline, the duration of diabetes was 8.9 ± 7.8 years. The duration of diabetes was longer in the EOSS-3 group as compared to the EOSS-2 group (11.4 ± 8.2 years vs. 6.8 ± 6.9 years, *p* = 0.002). Both groups were able to substantially reduce their insulin requirements (Table 3). However, only the EOSS-2 group were also able to significantly reduce their HbA1c (Table 3). The proportion of patients with T2DM and a HbA1c less than 7.0% did not differ significantly between EOSS groups initially or at 12 months, nor did the proportion change significantly within each EOSS group in the study period. Within this cohort with T2DM, initial and 12-month weight did not significantly differ between EOSS groups. There was no significant difference in medication requirements or HbA1c between EOSS groups at 12 months, although the greater insulin use in the EOSS-3 group at baseline remained significant at 12 months.

The difference in prevalence and medication burden for hypertension and dyslipidaemia also remained significant between the EOSS groups at 12 months. Only the EOSS-3 group saw significant improvement in total cholesterol and LDL.

## 4. Discussion

This study demonstrated that both the EOSS-2 and EOSS-3 groups lost a significant amount of weight at 12 months, with a greater proportion of the EOSS-3 group achieving 10% weight loss at 12 months. This was despite a higher prevalence of T2DM, hypertension and dyslipidaemia as well as greater medication burden including insulin treatment in this study. This is the first study in a publicly funded multidisciplinary weight management program in Australia where the EOSS has been used to classify all enrolled patients. This study demonstrated a high burden of disease in this program with over 97% of patients having an EOSS score of 2 or more. We also know that obesity and the accumulation of co-morbidities is associated with social disadvantage [32,33], which is consistent with the finding of lower employment in the EOSS-3 group.

The population of this multidisciplinary weight management program had a significantly higher co-morbidity burden than other reported populations, with approximately 40% of patients in the EOSS-3 group, compared to 3.9–15% in other studies [12,21,34,35]. This is likely due to the strict referral criteria of the program, which requires patients to have a BMI ≥ 40 and an obesity-related co-morbidity. These criteria effectively exclude patients with EOSS-0 or EOSS-1 from entering the program and are borne out of a scarcity of access to multidisciplinary weight management programs in Australia [15].

Patients in both the EOSS-2 group and EOSS-3 group lost significant and clinically meaningful amounts of weight over 12 months, which also translated into improvements in T2DM and its treatment. Given that these milestones are significant in reducing or managing risk of T2DM and ischaemic heart disease [5,36,37], it is reassuring that patients, who, by definition, already have end-organ complications of obesity, are able to achieve clinically significant weight loss. Interestingly, despite their higher co-morbidity burden and social disadvantage, a larger proportion of the EOSS-3 group (40% of the patients) was able to achieve 10% body weight loss at 12 months. This finding contrasts with previous data suggesting that while patients of all EOSS stages can attain similar weight loss, higher EOSS groups take longer periods of time to lose the same amount of weight [12]. This may possibly result from the multidisciplinary nature of the program, which included on site supervised exercise classes, and the attention provided to manage physical and mental health co-morbidities in these patients with higher EOSS scores [28]. We have also demonstrated that the presence of obstructive sleep apnoea as well as use of CPAP did not affect weight outcomes at 12 months in this cohort [30], nor did the presence of T2DM at baseline affect weight loss at 6 months [31].

The unadjusted results showed that the EOSS-3 group had a greater mean weight loss, and that a greater proportion lost >5% and >10% weight at 12 months. However, after adjustment for age, the significance was lost for the difference in mean weight loss and proportion that lost >5%. Previous studies have demonstrated that older individuals more readily lose weight on a weight loss program compared to younger individuals [38,39,40]. Patients in the EOSS-3 group who had T2DM also had longer duration of diabetes compared to the EOSS-2 group, in keeping with the older age in the EOSS-3 group. Another study also found that EOSS-3 patients were older, in line with our EOSS-3 patients being older at baseline [21]. It has also been suggested that there is a lower failure to follow-up rate in older age groups [41], although the follow-up rate was similar between EOSS groups in our study despite differences in mean age. This may signify patients with greater health needs remaining in the program.

Although the HbA1c reductions in the two EOSS groups did not reach statistical significance, the fact that >40% of patients in each group were able to achieve an HbA1c < 7.0% is reassuring. This is particularly encouraging, given that a recent study in the local diabetes service showed that less than a third of patients with T2DM and BMI ≥ 35 achieved an HbA1c < 7.0% [42]. The greater improvement in total and LDL cholesterol seen in the EOSS-3 group vs. the EOSS-2 group is interesting. Although the baseline cholesterol and LDL levels as well as the number of cholesterol-lowering medications were higher at baseline in the EOSS-3 group, the amount of cholesterol-lowering medication does not change over the 12 months. While it is known that weight loss can induce reductions in total cholesterol and LDL [43], the reduction in cholesterol in the EOSS-3 group are significantly greater than expected for the amount of weight loss, with no change in the EOSS-2 group. Although not explored, adherence to medication, may have been greater after starting the program, resulting in an improved lipid profile in the EOSS-3 group where prescribed cholesterol medication was higher at baseline. Adherence to cholesterol lowering medication has been shown to be poor, and previous systematic reviews and meta-analyses have shown the improved adherence as well as cholesterol levels with various interventions [44,45]. The regular follow-up in the weight management program itself could have served as reminders for patients to improve medication adherence.

Patients who are seeking bariatric surgery tend to have a higher risk of eating disorders [46]. It is therefore reassuring that we have recently shown that the eating disorder risk, psychological distress and quality of life improve over 12 months in a multidisciplinary weight management program, independent of weight loss [29], which may in turn help improve the psychological component of the EOSS score. This would also reduce the potential risk of people with eating disorders, if they did proceed to have bariatric surgery after having received multidisciplinary weight management.

There are several limitations of this study. This is a single centre study with a limited population size and dropouts. The patients were self-selecting, with strict entry criteria, and those that remained on the program may have been highly motivated. However, despite being a single centre study, the program consists of a multidisciplinary team including several physicians, and the drop-out rate is not different from other such weight management programs [12,41,47]. The referral criteria meant that very few patients had EOSS-0 or EOSS-1, so comparisons with those groups could not be made. The strength of this study is that all patients enrolled in the program were included in the study, and the EOSS classification was carried out by a single scorer, which provided consistency.

## 5. Conclusions

Patients attending this multidisciplinary intensive WMP had class 3 obesity and clinically significant and established weight-related complications. Despite the complexity of these patients, this service resulted in clinically meaningful weight loss at 12 months. Patients with T2DM also had improvements in glycaemic control with less insulin use regardless of their severity or duration of weight-related complications. Based on these real-world findings, intensive non-surgical multidisciplinary WMPs can result in weight loss in complex patients with class 3 obesity, including those with established complications. Our research will likely contribute to scalable and sustainable improvements in the standards of care for severe obesity and related complications in Australia’s health care system. 

## Figures and Tables

**Table 1:** EOSS definitions, from Canning et al. [12].

EOSS Stage	Stage Conceptual Description	Study Definition
0	No apparent obesity-related risk factors, physical symptoms, psychopathology, functional limitations and/or impairments of well-being.	No EOSS factors are reported.
1	Presence of obesity-related subclinical risk factors, mild physical symptoms, mild psychopathology, mild functional limitations and/or impairment of well-being.	Any of the following: Glucose ≥ 5.6 mmol/L; Cholesterol ≥ 5.2 mmol/L; Triglycerides ≥ 1.7 mmol/L; HDL ≤ 1.6 mmol/L; LDL ≥ 3.3 mmol/L; Systolic blood pressure ≥ 130 mmHg; Diastolic blood pressure ≥ 85 mmHg.
2	Presence of established obesity-related chronic disease, moderate limitations in activities of daily living and/or well-being.	Any of the following: Glucose ≥ 6.9 mmol/L; Diagnosed type 2 diabetes or type 2 diabetes medication; Cholesterol ≥ 6.2 mmol/L; Diagnosed hypercholesterolaemia; Triglycerides ≥ 2.2 mmol/L; HDL ≤ 1.0 mmol/L; LDL ≥ 4.1 mmol/L; Diagnosed hyperlipidaemia or hyperlipidaemia medication; Systolic blood pressure ≥ 140 mmHg; Diastolic blood pressure ≥ 90 mmHg; Diagnosed hypertension or hypertension medication; Sleep apnoea; Gout; Arthritis; Anxiety; Atherosclerosis; Fatty liver; Congestive heart failure medication; Blood thinner medication; Depression.
3	Established end-organ damage, significant psychopathology, significant functional limitations and/or impairment of well-being.	Any of the following: Angina; Heart attack; Heart failure; Thrombosis; Coronary artery disease; Coronary obstructive pulmonary disease; Dyspnoea; Exercise dyspnoea; Coronary artery bypass surgery; Stroke.
4	Severe (potentially end-stage) disabilities from obesity-related chronic diseases, disabling psychopathology, functional limitations and/or impairment of well-being.	No data on these factors are available to evaluate this stage.

**Table 2:** Comparison at baseline by initial EOSS.

	EOSS-2 (*n* = 101)	EOSS-3 (*n* = 71)	*p*-Value
Age (years)	46.5 ± 13.9	56.0 ± 11.6	<0.001 *
Sex (*n*, % Female)	77 (76.2%)	46 (64.8%)	0.101
Employed (*n*, %)	39 (38.6%)	16 (22.5%)	0.026 *
Weight (in kg)	141.2 ± 32.4	143.5 ± 28.4	0.471
BMI	50.4 ± 9.1	52.3 ± 8.6	0.065
Desiring bariatric surgery (*n*, % yes)	57 (56.4%)	41 (57.7%)	0.864
T2DM Outcomes (Patients with T2DM at baseline only)			
Number with T2DM (*n*, %)	50 (49.5%)	46 (64.8%)	0.047 *
Duration of diabetes	7.2 ± 7.0	11.7 ± 8.1	<0.001 *
HbA1c (%)	7.6 ± 1.7	7.6 ± 1.6	0.794
Patients with HbA1c < 7% (*n*, %)	21 (41.7%)	21 (45.6%)	0.848
Number of non-insulin agents	1.5 ± 0.9	1.5 ± 0.9	0.648
Patients requiring insulin (*n*, %)	11 (22.0%)	20 (43.5%)	0.025 *
Daily dose of insulin (units)	160.0 ± 134.4	122.5 ± 72.3	0.649
Hypertension			
Number with hypertension (*n*, %)	69 (68.3%)	59 (83.1%)	0.029 *
Number of blood pressure medications	1.0 ± 1.2	2.0 ± 1.4	<0.001 *
Systolic blood pressure (mmHg)	130.4 ± 15.1	132.6 ± 18.0	0.512
Dyslipidaemia			
Number with dyslipidaemia (*n*, %)	45 (44.6%)	47 (66.2%)	0.005 *
Number of cholesterol-lowering agents	0.4 ± 0.5	0.8 ± 0.7	<0.001 *
Total cholesterol (mmol/L)	4.4 ± 1.1	4.5 ± 1.0	0.568
LDL (mmol/L)	2.5 ± 1.0	2.3 ± 0.9	0.527
Triglycerides (mmol/L)	2.0 ± 1.3	1.8 ± 0.8	0.886
HDL (mmol/L)	1.1 ± 0.3	1.2 ± 0.3	0.160
Micronutrients			
Adjusted Calcium (mmol/L)	2.4 ± 0.1	2.4 ± 0.1	0.348
Magnesium (mmol/L)	0.8 ± 0.1	0.8 ± 0.1	0.434
Phosphate (mmol/L)	1.1 ± 0.2	1.1 ± 0.2	0.842
Iron (μmol/L)	12.5 ± 3.8	12.8 ± 4.3	0.766
Vitamin B12 (pmol/L)	296.1 ± 154.6	352.1 ± 230.4	0.158
Folate (nmol/L)	25.6 ± 8.8	25.6 ± 10.0	0.874
Vitamin D (nmol/L)	51.6 ± 24.7	58.4 ± 20.3	0.127

* Significant at *p* < 0.05. LDL: low density lipoprotein; HDL: high density lipoprotein; BMI: body mass index; T2DM: type 2 diabetes mellitus.

**Table 3:** Initial and 12-month outcomes by EOSS group.

	EOSS-2 (*n* = 63)			EOSS-3 (*n* = 45)		
	Initial	12 Months	*p*-Value	Initial	12 Months	*p*-Value
Weight (in kg)	139.4 ± 31.8	131.8 ± 31.8	<0.001 *	141.4 ± 24.2	129.8 ± 24.3	<0.001 *
BMI	50.3 ± 9.3	47.5 ± 9.4	<0.001 *	51.4 ± 8.1	47.2 ± 8.3	<0.001 *
T2DM (Patients with T2DM at baseline only)						
Number of patients with T2DM (*n*, %)	36 (57.1%)	32 (50.8%)	0.474	34 (75.6%)	32 (71.1%)	0.726
HbA1c (T2DM patients only) (%)	7.8 ± 1.8	7.2 ± 1.5	0.034 *	7.9 ± 1.6	7.4 ± 1.3	0.086
Percentage with HbA1c < 7% (*n*, %)	15 (41.7%)	17 (47.2%)	0.635	13 (38.2%)	14 (41.1%)	0.804
Number of non-insulin agents (T2DM patients only)	1.6 ± 0.9	1.7 ± 1.0	0.710	1.5 ± 1.0	1.6 ± 0.8	0.553
Percentage requiring insulin (T2DM patients only) (*n*, %)	9 (25.0%)	9 (25.0%)	1.000	17 (50.0%)	17 (50.0%)	1.000
Daily dose of insulin (T2DM patients only) (units)	161.1 ± 145.5	66.4 ± 40.3	0.037 *	111.8 ± 82.7	71.0 ± 66.6	0.007 *
Hypertension						
Percentage with hypertension (*n*, %)	49 (77.8%)	45 (71.4%)	0.412	38 (84.4%)	39 (86.7%)	0.764
Number of blood pressure medications	1.2 ± 1.2	1.1 ± 1.2	0.112	2.0 ± 1.3	1.9 ± 1.3	0.434
Systolic blood pressure (mmHg)	131.2 ± 15.9	133.0 ± 17.6	0.561	134.8 ± 18.6	128.6 ± 18.7	0.049 *
Dyslipidaemia						
Percentage with dyslipidaemia (*n*, %)	31 (49.2%)	32 (50.8%)	0.858	33 (73.3%)	35 (77.8%)	0.623
Number of cholesterol-lowering agents	0.5 ± 0.6	0.5 ± 0.6	0.414	0.9 ± 0.8	1.0 ± 0.7	0.157
Total cholesterol (mmol/L)	4.3 ± 1.0	4.4 ± 1.1	0.635	4.6 ± 1.0	3.8 ± 0.8	<0.001 *
LDL (mmol/L)	2.3 ± 0.9	2.3 ± 0.6	0.640	2.4 ± 0.9	1.8 ± 0.8	0.006 *
Triglycerides (mmol/L)	1.9 ± 1.4	1.9 ± 1.9	0.945	1.8 ± 0.8	2.1 ± 1.2	0.275
HDL (mmol/L)	1.1 ± 0.3	1.1 ± 0.3	0.904	1.2 ± 0.3	1.1 ± 0.2	0.099

* Significant at *p* < 0.05. LDL: low density lipoprotein. HDL: high density lipoprotein. BMI: body mass index. T2DM: type 2 diabetes mellitus.

**Table 4:** Comparison at 12 months by initial EOSS.

	EOSS-2	EOSS-3	*p*-Value
Number of patients with 12-month follow-up (*n*, %)	63 (62.4%)	45 (63.4%)	0.893
Weight loss (%)	5.7 ± 5.3	8.2 ± 7.3	0.047 *
Percentage who lost >5% weight (*n*, %)	33 (52.4%)	32 (71.1%)	0.050
Percentage who lost >10% weight (*n*, %)	10 (15.9%)	18 (40.0%)	0.005 *
T2DM Outcomes (Patients with T2DM at baseline only)			
Number with T2DM follow-up at 12 months (*n*, %)	32 (50.8%)	32 (71.1%)	0.034 *
Weight loss (%)	5.7 ± 6.0	7.7 ± 6.1	0.184
HbA1c at 12 months (%)	7.2 ± 1.5	7.4 ± 1.3	0.329
Percentage with HbA1c < 7% (*n*, %)	17 (47.2%)	14 (41.2%)	0.610
Change in HbA1c (in %)	0.6 ± 1.4	0.5 ± 1.4	0.917
Number of patients with remission of T2DM (*n*, %)	4 (11.1%)	2 (5.9%)	0.434
Number of non-insulin agents	1.7 ± 1.0	1.6 ± 0.8	0.980
Number requiring insulin (*n*, %)	9 (25%)	17 (50.0%)	0.030 *
Percentage of those initially on insulin who ceased insulin (*n*, %)	1 (11.1%)	1 (5.8%)	0.634
Daily dose of insulin (units)	66.4 ± 40.3	71.0 ± 66.6	0.619
Reduction in insulin dose (units)	94.7 ± 123.1	40.8 ± 55.8	0.234
Hypertension			
Number with hypertension (*n*, %)	45 (71.4%)	39 (86.7%)	0.060
Number of anti-hypertensives	1.1 ± 1.2	1.9 ± 1.3	<0.001 *
Systolic blood pressure (mmHg)	133.0 ± 17.6	128.6 ± 18.7	0.873
Dyslipidaemia			
Number with dyslipidaemia (*n*, %)	32 (50.8%)	35 (77.8%)	0.004 *
Number of cholesterol-lowering agents	0.5 ± 0.6	1.0 ± 0.7	0.001 *
Total cholesterol (mmol/L)	4.4 ± 1.1	3.8 ± 0.8	0.006 *
Change in cholesterol (mmol/L)	0.2 ± 1.4	-0.8 ± 1.1	0.001 *
LDL (mmol/L)	2.3 ± 0.6	1.8 ± 0.8	0.027 *
Triglycerides (mmol/L)	1.9 ± 1.8	2.1 ± 1.2	0.234
HDL (mmol/L)	1.1 ± 0.3	1.1 ± 0.2	0.266

* Significant at *p* < 0.05. LDL: low density lipoprotein; HDL: high density lipoprotein; BMI: body mass index; T2DM: type 2 diabetes mellitus.

## Data Availability

The data used to support the findings of this study are available from the corresponding author upon request.
